# Ni-Based SBA-15 Catalysts Modified with CeMnO_x_ for CO_2_ Valorization via Dry Reforming of Methane: Effect of Composition on Modulating Activity and H_2_/CO Ratio

**DOI:** 10.3390/nano13192641

**Published:** 2023-09-26

**Authors:** Maria V. Grabchenko, Natalia V. Dorofeeva, Valery A. Svetlichnyi, Yurii V. Larichev, Valeria La Parola, Leonarda Francesca Liotta, Sergei A. Kulinich, Olga V. Vodyankina

**Affiliations:** 1 Laboratory of Catalytic Research, Tomsk State University, 634050 Tomsk, Russia; 2 Laboratory of Advanced Materials and Technology, Siberian Physical Technical Institute, Tomsk State University, 634050 Tomsk, Russia; 3 Boreskov Institute of Catalysis SB RAS (BIC SB RAS), 630090 Novosibirsk, Russia; 4 Institute for the Study of Nanostructured Materials (ISMN), National Research Council (CNR), 90146 Palermo, Italy; 5 Research Institute of Science & Technology, Tokai University, Hiratsuka, Kanagawa 259-1292, Japan

**Keywords:** dry reforming of methane (DRM), CeO_2_–MnO_x_, Ni-based catalysts, SBA-15, SAXS, Raman spectra

## Abstract

Dry reforming of methane with ratio CH_4_/CO_2_ = 1 is studied using supported Ni catalysts on SBA-15 modified by CeMnO_x_ mixed oxides with different Ce/Mn ratios (0.25, 1 and 9). The obtained samples are characterized by wide-angle XRD, SAXS, N_2_ sorption, TPR-H_2_, TEM, UV–vis and Raman spectroscopies. The SBA-15 modification with CeMnO_x_ decreases the sizes of NiO nanoparticles and enhances the NiO–support interaction. When Ce/Mn = 9, the NiO forms small particles on the surface of large CeO_2_ particles and/or interacts with CeO_2_, forming mixed phases. The best catalytic performance (at 650 °C, CH_4_ and CO_2_ conversions are 51 and 69%, respectively) is achieved over the Ni/CeMnO_x_/SBA-15 (9:1) catalyst. The peculiar CeMnO_x_ composition (Ce/Mn = 9) also improves the catalyst stability: In a 24 h stability test, the CH_4_ conversion decreases by 18 rel.% as compared to a 30 rel.% decrease for unmodified catalyst. The enhanced catalytic stability of Ni/CeMnO_x_/SBA-15 (9:1) is attributed to the high concentration of reactive peroxo (O^−^) and superoxo (O_2_^−^) species that significantly lower the amount of coke in comparison with Ni-SBA-15 unmodified catalyst (weight loss of 2.7% vs. 42.2%). Ni-SBA-15 modified with equimolar Ce/Mn ratio or Mn excess is less performing. Ni/CeMnO_x_/SBA-15 (1:4) with the highest content of manganese shows the minimum conversions of reagents in the entire temperature range (X(CO_2_) = 4–36%, X(CH_4_) = 8–58%). This finding is possibly attributed to the presence of manganese oxide, which decorates the Ni particles due to its redistribution at the preparation stage.

## 1. Introduction

The growing energy needs of humankind, global warming, climate change and the necessity for rational use of fossil resources determine the growing worldwide interest in renewable energy sources. To reduce the dependence on fossil resources such as oil, coal, gas, and improve the environmental situation, it is necessary to elaborate new highly productive processes using alternative sources of energy. With population growth and the increase in energy consumption and quality of life, the CO_2_ emissions will continue to rise unless the current trend changes [1]. One of the most promising alternatives for natural gas valorization, involving CO_2_ recycling, is the process of dry methane reforming (DRM). In this process, the synthesis gas (H_2_/CO) is the main product, which is widely used in the production of electrical and thermal energy, in the process of the synthesis of methanol and the Fischer–Tropsch reaction. Thus, the DRM process using various catalysts contributes to the CO_2_ utilization and its transformation into value-added products.

High catalytic activity and availability are the main reasons which make Ni-based catalysts very promising for extensive investigations in the DRM process. However, two main drawbacks of supported nickel catalysts, including the sintering of active Ni nanoparticles (NPs) under high temperatures and high coke deposition, prevent its wide applications. By now, many efforts have been conducted to modify the chemical composition of supports or/and to promote the catalyst with additives, while the development of more stable Ni-based catalysts with high resistance to coke deposition and sintering is still required [2,3,4].

Recently, mesoporous ordered silica-based materials have been used to develop heterogeneous catalysts for application in the DRM process owing to their high specific surface area which is involved in generating (ultra)small metallic clusters with a homogeneous distribution and well-ordered structure [5]. However, the question about the catalytic behavior of Ni catalysts prepared on the basis of supports with well-ordered structure remains open. The Ni-based catalysts supported on mesoporous carriers, such as SBA-15, MCM-41, KCC-1, silicalite-1, zeolite [6,7,8,9,10], are well known for their high activity and resistance to coke formation in high-temperature reforming processes. Moreover, the stabilization of Ni metal species due to interaction with the support favors the distribution of active component as dispersed NPs and allows lowering of the amount of introduced metal. In ref. [7], a series of Ni catalysts were encapsulated by silicalite-1 zeolite in DRM. To improve the stability of the resulting catalysts, the authors used an encapsulation strategy. However, the intrinsic nature of the catalytic system also played an important role in preventing both coking and sintering of Ni NPs during the DRM.

Among such materials, silica-based SBA-15 has recently attracted particular attention because of its pore structure that consists of highly ordered 2D hexagonal pores with highly uniform porosity, thick pore walls and high specific surface area. This choice was also dictated by promising results demonstrated by Ni-based SBA-15-supported systems tested in DRM [11,12,13,14]. In ref. [6], it was shown that the Ni-SBA-15 exhibited the best stability under DRM conditions when compared with Ni-KIT-6 and Ni-MCM-41. In ref. [13], the authors concluded that the higher catalytic stability of the Ni/SBA-15 catalyst in DRM possibly depended on the existence of Ni-O-Si bonds. In ref. [11], the properties and catalytic activity of Ni/SBA-15 were studied as a function of the TEOS/P123 mass ratio. High catalytic performance of Ni/SBA-15 can be achieved due to strong metal–support interactions, and due to an optimal TEOS/P123 ratio with optimal textural properties consisting of a well-ordered hexagonal mesopores structure.

Nevertheless, at elevated temperatures, Ni NPs were reported to diffuse from tubular pore channels of an SBA-15 support onto the external surface owing to the weakening of Ni–support interactions during the catalysis process [15]. The use of oxide modifiers is thought to be a promising approach to solving this problem and to tune the interaction between silica support and Ni NPs. High catalytic performance in reforming processes can be improved by modifying Ni/SBA-15 with different oxides or its mixtures such as Ce [16,17], Y [18], Ce-Y [19], La [20], Zr and Mn [21,22]. To the best of our knowledge, there were no attempts to modify Ni/SBA-15 DRM systems with mixed CeMnO_x_ oxides to explore any possible synergism of Ce and Mn oxides and characterize the features and catalytic performance of the resulting composites, especially the effect of these modifiers on activity and stability in the DRM process.

Based on our previous studies that involved the DRM process over Ni catalysts supported on CeO_2_–MnO_x_ with varied Ce/Mn ratios [23], it can be suggested that the modifying of Ni/SBA-15 with CeO_2_–MnO_x_ oxides will lead to improved catalytic stability and resistance to carbon accumulation in DRM. This should result from a decrease in the acidity of such a composite catalyst, as well as because of an increase in oxygen reactivity and thermal stability of its well-ordered SBA-15 support.

In this work, the textural and morphological properties of modified and unmodified supports were studied by preparing nickel catalysts using SBA-15 and CeMnO_x_/SBA-15 with varied Ce/Mn ratios as support materials which were evaluated in gradient and long- run modes for the first time. Fresh and spent catalysts were studied systematically using a set of methods to disclose the role of the support material and its interaction with metallic NPs in catalytic activity and stability during the DRM reaction. Carbon deposits were studied qualitatively and quantitatively. The approaches described in the present work are expected to provide valuable insights for the selection of a suitable support when designing highly efficient and stable DRM catalysts.

## 2. Experiment

### 2.1. Preparation of Support and Catalysts

The initial SBA-15 support with a uniform pore size of 4.5–8.0 nm was prepared by the template technique using a Pluronic P123 triblock copolymer as surfactant (PEO20PPO70PEO20) [24]. For this, 16 g of P123 was dissolved in 500 mL of 2 M HCl solution and stirred at room temperature until transparent color of the mixture. The reaction mixture was transferred to an autoclave, and 34.28 g of tetraethylorthosilicate (TEOS) was added. The reagents were stirred at room temperature for 20 h. Then, the product was aged at 100 °C for 48 h. The resulting product was filtered and repeatedly washed with water and then dried stepwise as follows: for 48 h at room temperature, for 2 h at 100 °C and for 3 h at 120 °C. The prepared sample was calcined at 500 °C with a heating rate of 1 °C/min for 5 h.

The SBA-15 surface was modified by binary oxides of Ce and Mn using sequential wetness impregnation with an aqueous solution of cerium and manganese nitrates (Ce(NO_3_)_3_·6H_2_O and Mn(NO_3_)_2·_6H_2_O) with the addition of a citric acid solution (C_6_H_8_O_7_) in the ratio of C_6_H_8_O_7_/(Ce + Mn) = 2. The prepared materials were dried at 120 °C and calcined at 500 °C with a heating rate of 1 °C/min for 5 h. The molar ratios of Ce/Mn in CeMnO_x_ modified SBA-15 varied within 0.25, 1 and 9 and these supports were designated as CeMnO_x_/SBA-15 (1:4), CeMnO_x_/SBA-15 (1:1) and CeMnO_x_/ SBA-15 (9:1), respectively.

The Ni-containing catalysts based on the unmodified and modified supports obtained were prepared by incipient wetness impregnation using an aqueous solution of Ni(NO_3_)_2_·6H_2_O. The nominal nickel loading was 10 wt.%. The catalyst samples were dried at 120 °C and calcined in a static mode at 800 °C with rate of 5 °C/min for 3 h. The resulting catalysts were labeled as follows: Ni/SBA-15, Ni/CeMnO_x_/SBA-15 (9:1), Ni/CeMnO_x_/SBA-15 (1:1) and Ni/CeMnO_x_/SBA-15 (1:4).

### 2.2. Characterization of Materials

X-ray fluorescence analysis (XRF) was used for the elemental analysis of prepared samples (XRF-1800, Shimadzu, Kyoto, Japan).

Textural properties (specific surface area, pore volume and pore size) of samples were measured using the 3Flex gas adsorption analyzer (Micromeritics, Norcross, GA, USA) by the low-temperature N_2_ sorption method. Each sample was preliminarily degassed under vacuum at 200 °C for 2 h. The pore distribution was calculated from the desorption branch of isotherm by the BJH method.

The structural studies of the prepared materials were carried out by means of the small-angle X-ray scattering (SAXS) method using a S3 MICRO tool (HECUS, Austria) with point collimation and Cu radiation at 50 W. The SAXS patterns were registered in the q range of 0.01–0.6 Å^–1^ (q = 4π sinθ/λ). A detailed description of the contrasting technique for excluding the residual scattering factor from the support was previously reported elsewhere [25]. In the present work, the residual scattering signal was attributed to particles of a heavier phase than SiO_2_. The ATSAS software package [26] was used to process the experimental data.

Wide-angle X-ray diffraction (XRD) patterns were registered by XRD-7000 (Shimadzu, Kyoto, Japan). The composition of the crystalline phase was determined using the JCPDS database and POWDER CELL 2.4 software.

The redox properties of the prepared samples were studied using a temperature-programmed reduction (H_2_-TPR) method using the Autochem 2950 (Micromeritics, Norcross, GA, USA). A cooled trap (with temperature −86 °C) was used to remove water. Before each TPR-H_2_ experiment, the samples were pre-oxidized at 750 °C, while the temperature range during the H_2_-TPR experiments was from 25 to 900 °C.

The UV-vis diffuse reflectance spectroscopy (UV-vis DRS) was carried out on a Cary 100 spectrophotometer (Agilent Technologies, California, USA) with a DRA-CA-30I Labsphere attachment in the range of 230–800 nm with MgO as a reference.

Raman spectra were measured on an InVia confocal Raman microscope (Renishaw, Wotton-under-Edge, UK) equipped with a DM 2500M microscope (Leica, Wetzlar, Germany) with excitation at 532 nm.

The changes in morphology and structure of catalysts after undergoing reduction treatment prior to DRM tests, as well as the alterations in the used samples post-catalytic testing, were analyzed using transmission electron microscopy (TEM) including high-resolution TEM (HRTEM) and high-angle annular dark-field scanning transmission electron microscopy (HAADF-STEM) on a Themis Z (Thermo Fisher Scientific, Eindhoven, The Netherlands).

The quantification of deposited carbon on the surface of spent catalysts was carried out by the TGA method in air flow on a STAR tool (Mettler Toledo, Switzerland) equipped with a mass spectrometer (Balzers Quadstar) in order to confirm the CO_2_ evolution due to carbon oxidation.

### 2.3. Catalytic Test

The tests of dry reforming of methane (DRM) were performed in two modes: temperature gradient tests in the range of 450 to 800 °C and long-run stability tests at 650 °C for 24 h time on stream (ToS). The Ni/CeMnO_x_/SBA-15 (9:1) catalyst was additionally tested for stability during 80 h ToS. The description of equipment used, as well as experimental conditions and calculation of CH_4_/CO_2_ conversion and product selectivity can be found elsewhere [23]. Before the reaction, the catalysts were pretreated in situ in an O_2_ stream (5 vol % in He, 50 mL/min) at 350 °C for 0.5 h. After cooling to room temperature, the samples were reduced in an H_2_ stream (5 vol % in He, 30 mL/min), while increasing the temperature to 750 °C at a linear heating rate of 10 °C/min and a holding time of 1 h.

## 3. Results and Discussion

### 3.1. Characterization of Calcined Supports and Catalysts

#### 3.1.1. X-ray Fluorescence Analysis (XRF)

The elemental composition (wt %) in terms of oxides (with oxygen content) of the prepared samples, determined by the XRF, is shown in Table 1. The real content agrees with the nominal one.

#### 3.1.2. BET Surface Area and Pore Structure

**Figure 1:**
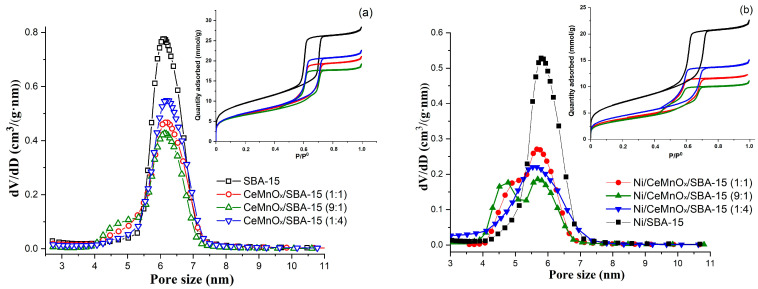
Pore size distributions and adsorption–desorption isotherms for the calcined supports (**a**) and Ni catalysts (**b**).

The textural properties of the prepared samples were characterized by N_2_ adsorption-desorption and are summarized in Figure 1 and Table 2. The nitrogen adsorption-desorption isotherms for initial SBA-15 support correspond to type IV with a hysteresis of H1 type (IUPAC) (insert in Figure 1a). The shape of the hysteresis loop at relative pressures of 0.54–0.7 indicates the mesoporous structure of the support with a cylindrical pore geometry. The isotherms of CeMnO_x_-modified supports and NiO loaded catalysts retain the same shape, while the quantity adsorbed is lowered and the capillary condensation step is shifted towards lower relative pressures (from 0.54 to 0.45).

The SBA-15 support is characterized by a narrow pore size distribution in the range of 5.2 to 7 nm with a maximum at 6.1 nm typical of an ordered silica structure (Figure 1a). The high values of specific surface area (763 m^2^/g) and pore volume (0.98 cm^3^/g) of initial SBA-15 support significantly decrease after impregnation by Ce and Mn nitrate solutions whereas the pore size remains almost unchanged, indicating that most of oxide components have been introduced into the pore channels (Table 1 and Figure 1a) [27].

After impregnation of the SBA-15 with nickel nitrate solution (Ni/SBA-15), the specific surface area and pore volume decrease to up to 494 m^2^/g and 0.7 cm^3^/g, respectively. The maximum of the pore size distribution shifts from 6 to 5.8 nm, which indicates the filling of the porous channels of the SBA-15 support by NiO particles (Figure 1b). The impregnation of CeMnO_x_/SBA-15 supports by Ni nitrate solutions also leads to a decrease in the specific surface area and pore volume. For the Ni/CeMnO_x_/SBA-15 (9:1) and Ni/CeMnO_x_/SBA-15 (1:1) catalysts, the peaks of pore size distribution are slightly widened due to a partial blockage of the primary mesopores and formation of small mesopores with sizes of 4.8–5 nm. Such bimodal pore size distribution may be a result of formation of Ni-containing particles outside the SBA-15 porous matrix. For the Ni/CeMnO_x_/SBA-15 (1:4) sample, a pore size distribution was observed with a wide region from 4 to 7 nm, which may indicate a predominant distribution of both modifying oxides and nickel oxide particles mainly inside the support channels.

#### 3.1.3. Small-Angle X-ray Scattering (SAXS) and Wide-Angle X-ray Diffraction (XRD)

Figure S1 shows the SAXS patterns for SBA-15. For the initial SBA-15 sample, its small-angle reflections attributed to the (100), (110) and (200) planes indicate the presence of ordered mesopores with a body-centered cubic symmetry (Im3m). To study the effect of heat treatment on the change in the SBA-15 framework structure, an additional annealing at 800 °C was carried out. Calcination of SBA-15 at 800 °C for 3 h leads to a significant shift of the reflections and a decrease in the lattice parameter (Figure S1), which is explained by a partial degradation of the porous structure during calcination.

In Table 1 the structural parameters (d(100) and (*a*)) calculated by SAXS analysis of the SBA-15-based materials and supported Ni catalysts are listed. The introduction of cerium and manganese oxides into the SBA-15 matrix after calcination at 500 °C leads to a negligible decrease in the lattice parameter (*a*) typical of SBA-15 pores with a hexagonal structure (10.72 vs. ~10.5). Moreover, the plane distance d(100) of CeMn modified supports was slightly lower, confirming that the long-range order of SBA-15 was maintained.

Conversely, the subsequent Ni precursor introduction into the system with calcination at 800 °C leads to a small decrease in the hexagonal lattice parameter, likely due to both effects, higher calcination temperature and Ni introduction. Similarly, the d(100) value of all Ni catalysts were lower than those for the corresponding supports.

Nevertheless, the introduction of these additives leads to the stabilization of the SBA-15 porous matrix, since the calcination of the initial SBA-15 support at 800 °C results in a significantly greater decrease in the lattice parameter (*a* = 9.53 nm) compared with the cases of introduced additives after calcination under similar conditions (*a* = 10.12–10.24 nm).

**Figure 2:**
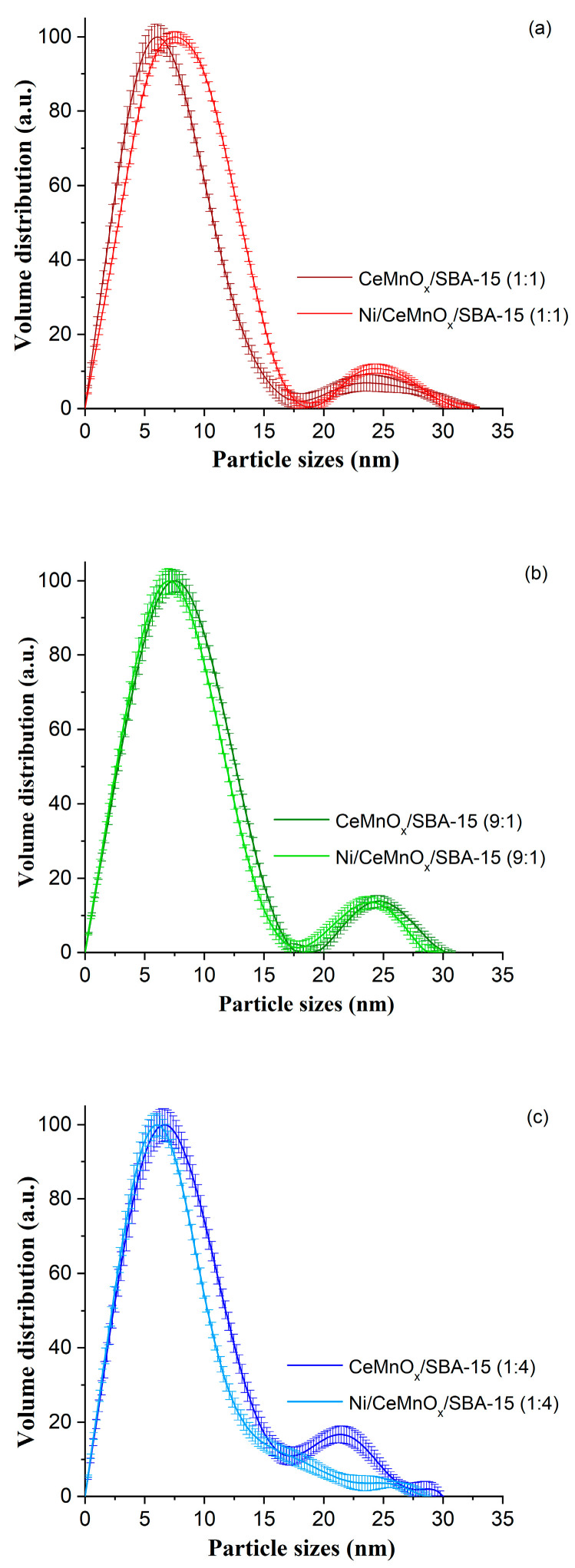
Particle size distribution for the corresponding calcined support/catalyst pair based on SAXS data: CeMnO_x_/SBA-15 (1:1) and Ni/CeMnO_x_/SBA-15 (1:1) (**a**), CeMnO_x_/SBA-15 (9:1) and Ni/CeMnO_x_/SBA-15 (9:1) (**b**), CeMnO_x_/SBA-15 (1:4) and Ni/CeMnO_x_/SBA-15 (1:4) (**c**).

To study the sizes of additive particles deposited on the SBA-15 matrix, a contrasting technique was used, which made it possible to identify phases with higher density as compared to silica. At the same time, the technique used did not allow one to distinguish particles of CeO_2_ from MnO_x_. Figure 2 shows the obtained volume size distributions of MnO_x_, CeO_2_ and NiO particles for the corresponding support/catalyst pair. The distributions are bimodal for all supports: the fraction with smaller sizes refers to particles present inside the SBA-15 pores, while the fraction with large sizes can be attributed to particles localized on the outer surface of the porous matrix. Taking into account the fact that the Ce/Mn ratio in the supports is different, it can be assumed that MnO_x_ particles form smaller particles on the outer support surface in comparison with CeO_2_. The sample enriched with manganese oxide (CeMnO_x_/SBA-15 (1:4)) is characterized by smaller particles located on the external surface of the support matrix (Figure 2c). For the sample enriched with cerium oxide (CeMnO_x_/SBA-15 (9:1)), the opposite picture is observed. For particles located inside the pores, there is no significant dependence of size on the type of oxide used (Figure 2b).

After adding nickel precursor to CeMnO_x_/SBA-15 (1:1) support, the changes in the distribution become visible, nickel oxide particles are localized in the range from 5 to 15 nm (Figure 2a). In the case of the CeMnO_x_/SBA-15 (9:1)–Ni/CeMnO_x_/SBA-15 (9:1) pair, both distributions are almost identical (Figure 2b). When comparing the CeMnO_x_/SBA-15 (1:4)–Ni/CeMnO_x_/SBA-15 (1:4) pair, the introduction of nickel precursor leads to smaller particle size of modifier oxides (Figure 2c) due to their redistribution between the outer surface and inside the pores of the support matrix.

Thus, we can assume a few types of interactions between the modified supports and active component. When CeO_2_ predominantly exists in the SBA-15 matrix, NiO forms small particles on the surface of large particles of CeO_2_ or/and NiO interacts with CeO_2_, forming mixed phases. Therefore, the distributions only slightly differ from each other, and the formation of NiO particles with other sizes was not observed (CeMnO_x_/SBA-15 (9:1)–Ni/CeMnO_x_/SBA-15 (9:1) samples). In the case of the predominant presence of MnO_x_ in the SBA-15 matrix, NiO is most likely introduced into the structure of manganese oxide particles. When comparing distributions for the CeMnO_x_/SBA-15 (1:4)–Ni/CeMnO_x_/SBA-15 (1:4) samples, it can be assumed that the large MnO_x_ particles are redispersed. With an equal ratio of CeO_2_ and MnO_x_ in the SBA-15 matrix, they interact more strongly with each other and, probably, NiO particles are formed separately (CeMnO_x_/SBA-15 (1:1)–Ni/CeMnO_x_/SBA-15 (1:1) samples).

**Figure 3:**
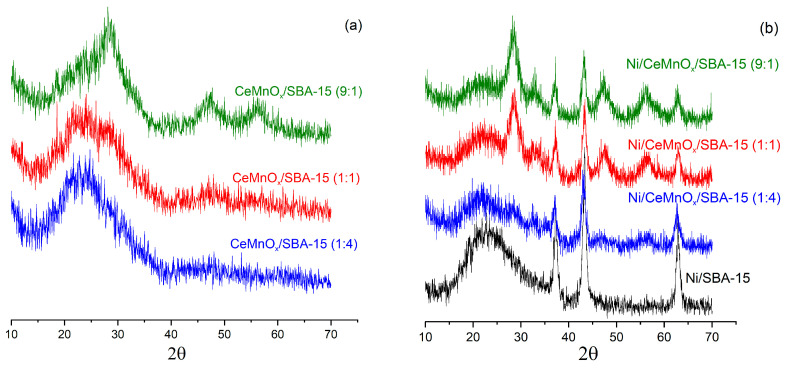
XRD patterns for calcined supports (**a**) and Ni catalysts (**b**).

The crystalline structures and particle sizes were determined by wide-angle XRD. Figure 3a,b shows the XRD patterns for the calcined supports and catalysts. The wide peak of amorphous silica located at ~10–35° is attributed to silica frameworks that belong to SBA-15 support for all samples.

The characteristic peaks of crystallites of cerium and manganese oxides are not detected for practically all supports, implying that the Ce and Mn species were incorporated into the mesoporous matrix of SBA-15 and/or were homogenously dispersed (Figure 3a). Only for a support with the maximal ceria loading (CeMnO_x_/SBA-15 (9:1) sample) were the peaks around 2θ = 47.2° and 56.5°, corresponding to plane (200) and (311) from CeO_2_, observed.

Figure 3b shows the diffraction patterns for the calcined catalysts. The reflections around 37, 43 and 62° are attributed to the NiO phase (JCPDS#47-1049). After Ni deposition on the supports, an increase in the intensity of the CeO_2_ reflections is observed, which can be related to higher calcination temperature in comparison with modified support. Moreover, the characteristic reflections of CeO_2_ shifted slightly towards higher angles: 2θ = 28.44, 32.81, 47.40 and 56.22 for Ni/CeMnO_x_/SBA-15 (9:1); 2θ = 28.54, 33.37, 47.51 and 56.50 for Ni/CeMnO_x_/SBA-15 (1:1) and 2θ = 28.70, 32.86, 47.43 and 56.20 for Ni/CeMnO_x_/SBA-15 (1:4).

This indicates that, during the impregnation step, a part of nickel cations is introduced into the CeMnO_x_ modifier to yield complex structures: Ce_1−(*x*+*y*)_Mn*_x_*Ni*_y_*O_2−δ_ and/or Ce_1−*y*_Ni*_y_*O_2−δ_ in the case of high content of CeO_2_; Ce_1−(*x*+*y*)_Mn*_x_*Ni*_y_*O_2−δ_ and/or Mn_1−*y*_Ni*_y_*O*_x_*in the case of high content of MnO_x_ [22].

Addition of MnO_x_ and CeO_2_ to Ni/SBA-15 was found to have a strong effect on the size of NiO crystallites. Thus, for catalysts based on binary oxide supports CeMnO_x_/SBA-15 the size varies from 8 to 10 nm, while the catalyst based on unmodified SBA-15 is characterized by the NiO size of 18 nm. The obtained data are consistent with the SAXS results.

#### 3.1.4. UV-Vis Diffuse Reflectance Spectroscopy (UV-Vis DRS) and Raman Spectroscopy

UV-vis DRS was used to identify the chemical environment and coordination structure of metal ions in the matrix of silica. The Kubelka–Munk functions of the corresponding bands are presented as a function of wavelength (Figure S2). The CeMnO_x_/SBA-15 (9:1) support shows a strong absorption peak around 350 nm. With an increase in the amount of manganese oxide, a red shift of this signal from 350 to 367 nm and the appearance of a band at about 525 nm are observed. According to refs. [28,29,30,31], the signal at about 270–340 nm corresponds to the O^2−^ → Ce^4+^ charge transfer transition in CeO_2_. The position of the absorption maximum depends on the presence of Ce^3+^, as well as on the morphologic characteristics of particles. The shift of the band maximum in the CeMnO_x_/SBA-15 series is caused by a slight doping of ceria with manganese oxide [32,33], which may indicate the presence of common boundaries of modifying oxide particles. Absorption observed in the range of 450–650 nm increases with a decrease in the Ce/Mn ratio, which is associated with the formation of X-ray amorphous manganese oxide Mn_2_O_3_ [34] or MnO_2_ [35] oxide particles.

The line in the range from 220 to 400 nm was detected for unmodified catalyst (Ni/SBA-15) and the sample with high ceria content (Ni/CeMnO_x_/SBA-15 (9:1)), which is associated with the charge transfer of octahedral Ni^2+^ species in NiO (Figure S2b) [36,37]. For the samples Ni/CeMnO_x_/SBA-15 (1:4) and Ni/CeMnO_x_/SBA-15 (1:1), the wide bands in the region of 324–720 nm with a maximum of ~460 nm can be assigned to octahedrally coordinated Ni species or isolated NiO on the external surface of the SBA-15 matrix. For the Ni/CeMnO_x_/SBA-15 (1:4) and Ni/CeMnO_x_/SBA-15 (1:1) catalysts, the wide band in the wavelength regions of 350–720 nm with a maximum at 460 nm was observed.

**Figure 4:**
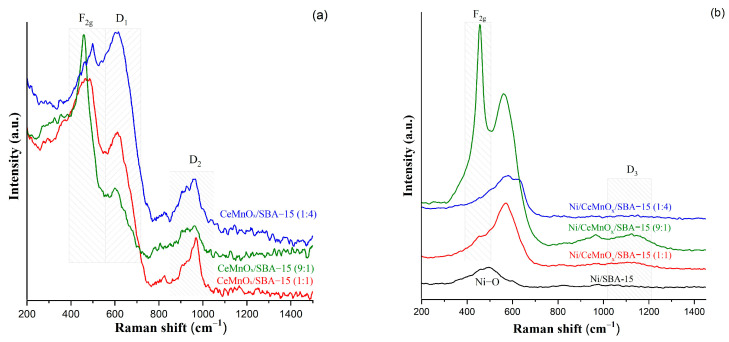
Raman spectra for the calcined supports (**a**) and Ni catalysts (**b**).

Figure 4 exhibits the Raman spectra of supports and Ni-containing catalysts. The peak observed at 462 cm^−1^ corresponds to the fluorite structure of ceria and is due to the Raman active F2g mode. This peak is especially intense for the sample with a high content of ceria (Ce/Mn = 9). In the spectrum for the Ni/CeMnO_x_/SBA-15 (1:1) sample, this band becomes wider, which indicates a more defective lattice structure. For the sample with the highest MnO_x_ content, this Raman band is not observed, but there is another one at 497 cm^−1^ indicating Mn-O-Mn vibrations.

For all modified supports, the peaks seen at 500–600 cm^−1^ and labeled as D1 are attributed to the presence of oxygen vacancies in the CeO_2_ structure. According to XRD data, the formation of disorder in the CeO_2_ structure is possible due to the replacement of the cerium ion by Mn^n+^ and the formation of Ce^3+^. A broad peak at ~960 cm^−1^ (D_2_) is related to the vibrations of peroxo sites (O^−^) [38,39,40].

The Raman spectra for Ni-containing catalysts (Figure 4b) show the broad bands at 500–600 cm^−1^ corresponding to Ni-O valence bonding modes (first-order phonons). For the Ni/SBA-15 system, this signal located at 500 cm^–1^ indicates the formation of bulk NiO. For catalysts based on modified SBA-15, this band is shifted towards higher frequencies (about 570 cm^−1^), which indicates strong interactions between the support surface and NiO particles. The bands in the region of 500–650 cm^−1^ also indicate the appearance of additional defects after the introduction of nickel precursor and include two components. The band at ~547 cm^−1^ is referred to the oxygen vacancy, which can be formed when Ce^4+^ is replaced by Ni^2+^ and Si^δ+^, and the mode at 615 cm^−1^ arises due to the oxygen vacancy formed as a result of Ce^3+^-Ce^4+^ transitions [41]. It is noteworthy that, for the Ni/CeMnO_x_/SBA-15 (1:1) and Ni/CeMnO_x_/SBA-15 (9:1) catalysts, new weak broad signals (D3) appear in the range of 1100–1200 cm^−1^, which can be explained by ν(O=O) stretching vibrations of superoxo species (O_2_^−^) [39,40].

### 3.2. H_2_-TPR Profiles and Structural Characterization of Reduced Catalysts

**Figure 5:**
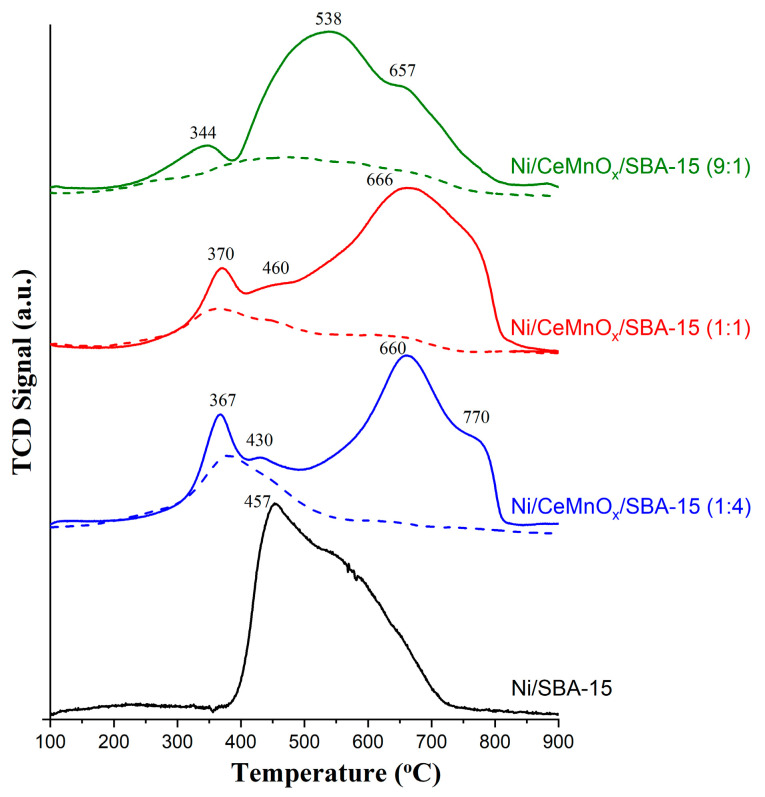
TPR-H_2_ profiles for the calcined supports (dash dotted lines) and Ni catalysts (solid lines).

Figure 5 exhibits the H_2_-TPR profiles for the calcined modified supports with different Ce/Mn molar ratios and the corresponding Ni/SBA-15 and Ni/CeMnO_x_/SBA-15 catalysts.

The H_2_ consumption by the CeMnO_x_/SBA-15 (9:1) support observed in the range of 240–750 °C is associated with the reduction of both the surface and bulk of ceria particles [42]. With an increase in the Mn content, for Ce/Mn ratios of 1:1 and 1:4, features of reduction of manganese ions Mn^4+^/Mn^3+^ → Mn^2+^ increases in the range of temperatures 290–525 °C and 280–570 °C. The reduction peaks seen at low temperature can be related to the reduction of fine particles of manganese oxide MnO_x_ [38]. The amount of H_2_ consumed for the total reduction of modifier oxides (Ce^4+^ → Ce_3+_ and Mn^4+^/Mn^3+^ → Mn^2+^) is ranging between 0.38 and 0.47 mmol/g (Table 3).

Reduction of NiO in Ni/SBA-15 occurs in the range of 400–700 °C (Figure 5). In ref. [43,44], the formation of NiO particles with different interaction with the SBA-15 surface was reported, which led to an increase in their reduction temperature compared to individual NiO. Thus, according to the conditional classification [43], NiO particles that moderately interact with their support (β-stage) are reduced from 390 °C to 510 °C. Then, particles that strongly interact with their support are reduced up to 730 °C (γ-stage). A significant contribution of the high-temperature component (510–730 °C) can also be associated with the encapsulation of NiO particles in SBA-15 mesopores. By comparing the experimental hydrogen consumption with the theoretical values calculated on the basis of the real Ni loading of all catalysts, the fraction of reduced nickel was determined. Such a value for Ni/SBA-15 is ~0.80.

When modifiers are introduced into the support, a negligible amount of weakly bound NiO particles that are reduced at temperatures of 300–400 °C are formed on its surface. It is noteworthy that, for Ni/CeMnO_x_/SBA-15 (9:1), the hydrogen consumption begins at a temperature of 230 °C, which indicates the formation of a Ce_1−*y*_Ni*_y_*O_2−δ_ or Ce_1−(*x*+*y*)_Mn*_x_*Ni*_y_*O_2−δ_ solid solution [22,45]. At the same time, the fraction of dispersed and strongly bound particles also increases as indicated by the shift of maxima in the range of 510–730 °C. For the Ni/CeMnO_x_/SBA-15 (9:1) catalyst, the Ni^2+^ reduction maximum shifts by 80 °C, while for Ni/CeMnO_x_/SBA-15 (1:1) and Ni/CeMnO_x_/SBA-15 (1:4) the shift of the maximum in the TPR-H_2_ profile is above 200 °C. Thus, the catalysts modified with CeO_2_ and MnO_x_ feature higher NiO dispersion compared to Ni/SBA-15, which agrees well with the XRD data (Table 2).

The fraction of Ni^0^ NPs reduced for Ni/CeMnO_x_/SBA-15 (9:1) and Ni/CeMnO_x_/SBA-15 (1:4) corresponds to 0.69, while for Ni/CeMnO_x_/SBA-15 (1:1) it is 0.93. The completeness of the reduction of active component is known to depend on its availability for the reducing agent after high-temperature calcination at the stage of catalyst preparation. In addition, a decrease in the degree of Ni^2+^ reduction in the presence of modifiers can be due to their redistribution and the formation of strong interaction with the active component, decoration of particles with oxide modifiers, and blocking in SBA-15 pores.

According to the SAXS data for calcined Ni/CeMnO_x_/SBA-15 (1:4) catalyst, there is a decrease in the size of deposited oxide particles compared to the size of the corresponding support (Figure 2c). A comparison of the distributions for the support and catalyst particles confirms the incorporation of Ni into the structure of manganese oxide particles, which leads to their dispersion.

According to TEM characterization (Figure S3), performed in order to study structural evolution of catalysts after reduction treatment at 750 °C (before catalytic test), in the Ni/CeMnO_x_/SBA-15 (1:4) the manganese is distributed around Ni particles with sizes of 15–40 nm, while smaller particles of ~3 nm surrounded by a modifier are also observed. A decrease in oxide particle size, according to the SAXS data, was observed also for Ni/CeMnO_x_/SBA-15 (9:1), however, to a lesser extent. For Ni/CeMnO_x_/SBA-15 (1:1), a broadening in the particle distribution from 5 to 15 nm occurs (Figure 2a), which indicates that the introduction of the active component does not lead to a redistribution of the modifier oxides. This is probably because the Ce/Mn ratio does not contribute to the formation of a solid solution on the surface, while the oxides are distributed in the form of individual CeO_2_ and MnO_x_ particles, which favor the NiO dispersion and do not decrease their reducibility.

**Figure 6:**
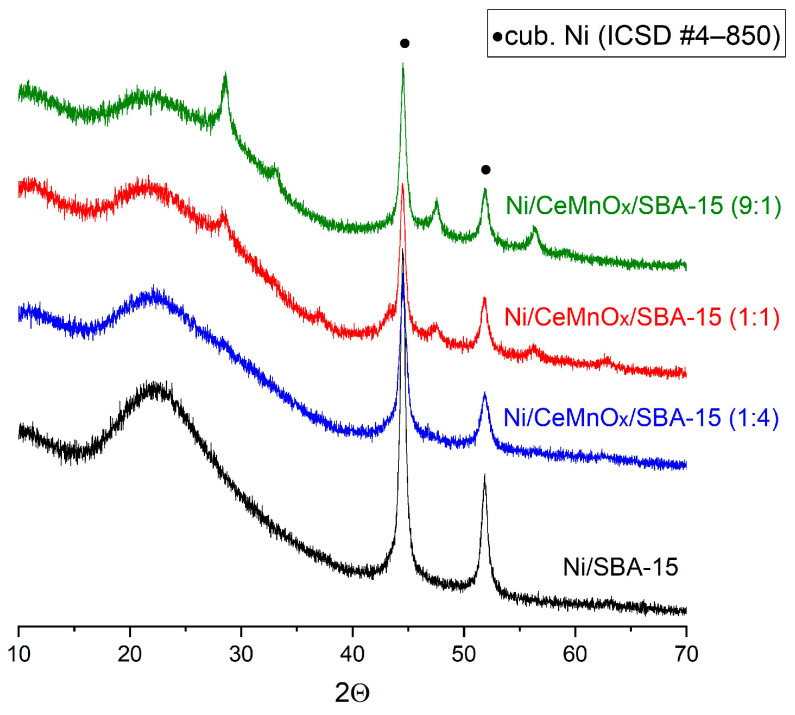
XRD patterns for the reduced Ni catalysts.

The size of Ni particles in the catalysts reduced in H_2_ stream (5 vol % in He) at 750 °C was determined by XRD data using the Debye–Scherrer equation. Figure 6 shows the diffraction patterns for the reduced catalysts. The Ni^0^ particle sizes increase from 13 to 19 nm in the series Ni/CeMnO_x_/SBA-15 (4:1) < Ni/CeMnO_x_/SBA-15 (1:1) ≈ Ni/CeMnO_x_/SBA-15 (9:1) < Ni/SBA-15 (Table 2). Probably, the formation of Ni NPs in the modified samples comparable in size to Ni/SBA-15 is associated with the presence of weakly interacting NiO particles (Figure 6) located on the outer surface of the SBA-15 matrix.

According to the SAXS data (Figure S4), by comparing calcined and reduced Ni/CeMnO_x_/SBA-15 (1:1) and Ni/SBA-15 samples we can see that the particle distribution maximum shifts to larger sizes but the increase in the Ni particles in SBA-15 pores is limited by the pore sizes. Therefore, significant changes in the distribution of particles in the range of 10–40 nm are associated with an increase in the particles localized on the outer surface of the support.

### 3.3. Catalyst Performance

#### 3.3.1. Gradient Catalytic Tests

**Figure 7:**
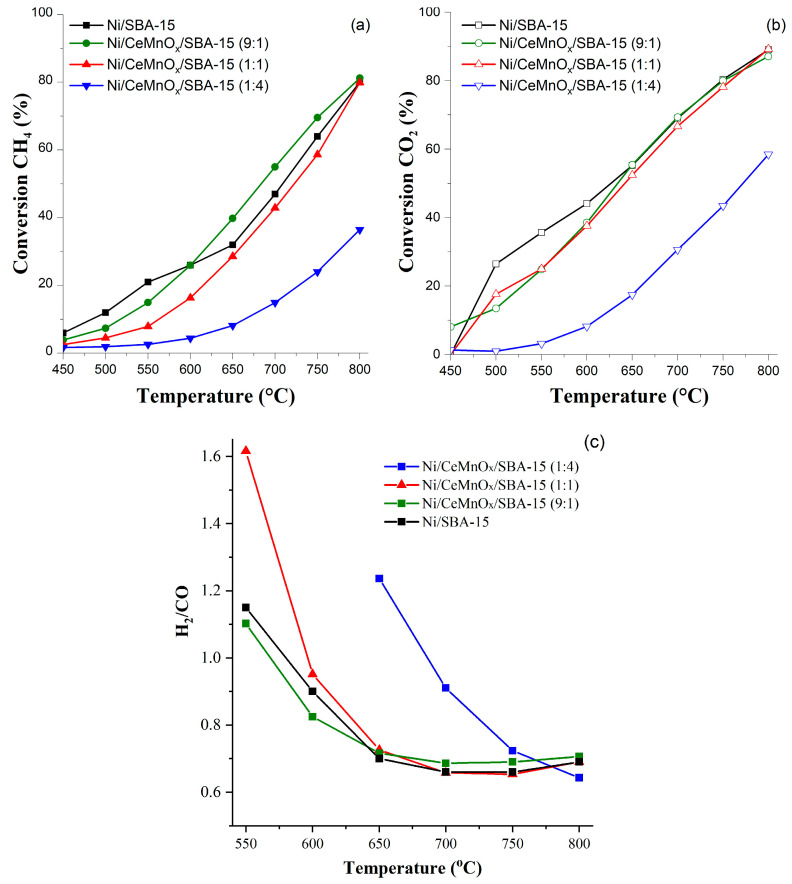
DRM results as a function of temperature at 450–800 °C: CH_4_ conversion (**a**), CO_2_ conversion (**b**), H_2_/CO ratio (**c**).

Figure 7 and Table 4 show the results of catalytic studies of the synthesized Ni/CeMnO_x_/SBA-15 samples in the DRM reaction. In the range of 450–600 °C, the conversion of the reagents increases in the series Ni/CeMnO_x_/SBA-15 (1:4) < Ni/CeMnO_x_/SBA-15 (1:1) < Ni/CeMnO_x_/SBA-15 (9:1) < Ni/SBA-15, and the X(CO_2_)/X(CH_4_) ratios are within 1.5–2.4. Above 650 °C, the CO_2_ and CH_4_ conversions on Ni/SBA-15, Ni/CeMnO_x_/SBA-15 (1:1) and Ni/CeMnO_x_/SBA-15 (9:1) catalysts are close and reach 80 and 90% at 800 °C, respectively.

The H_2_/CO ratio on the catalysts without modifiers and in the presence of additives with the ratios of 9:1 and 1:1 is about 0.7. Higher values of CO_2_ conversion compared to those of CH_4_ as well as H_2_/CO < 1 are associated with the reverse water-gas shift reaction CO_2_ + H_2_ ↔ CO + H_2_O. An increase in the process temperature also leads to the carbon oxidation via the reverse Boudouard reaction C + CO_2_ ↔ 2CO and an increase in CO concentration in the products. The catalyst with the highest content of manganese Ni/CeMnO_x_/SBA-15 (1:4) shows the minimum conversions of reagents over the entire temperature range compared to other catalysts (X(CO_2_) = 4–36%, X(CH_4_) = 8–58%). Probably, the low conversion of reactants for this catalyst is caused by decoration of Ni particles with manganese oxide due to its redistribution at the preparation stage, which is confirmed by the SAXS and TPD data. The H_2_/CO ratios close to 1 are achieved on the unmodified catalyst and on samples with Ce/Mn additives with the ratios of 1/1 and 9/1 in the temperature range of 550–600 °C, and for the sample with Ce/Mn = 1:4 it occurs at higher temperatures (650–700 °C).

#### 3.3.2. Long-Run Tests

**Figure 8:**
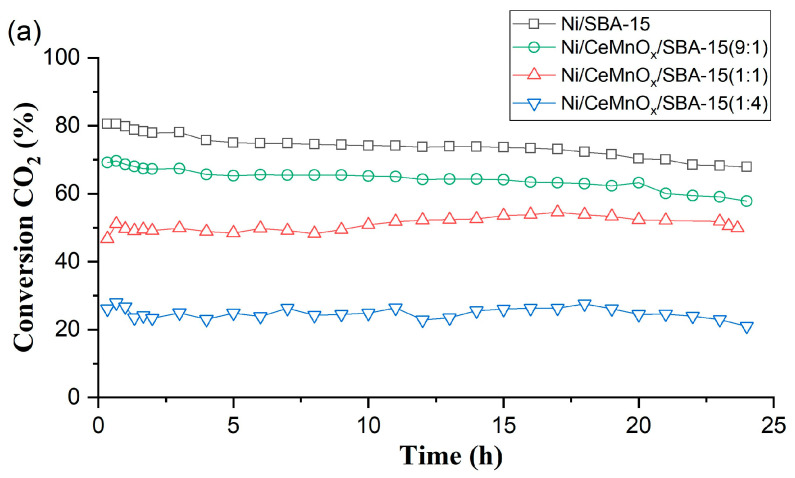

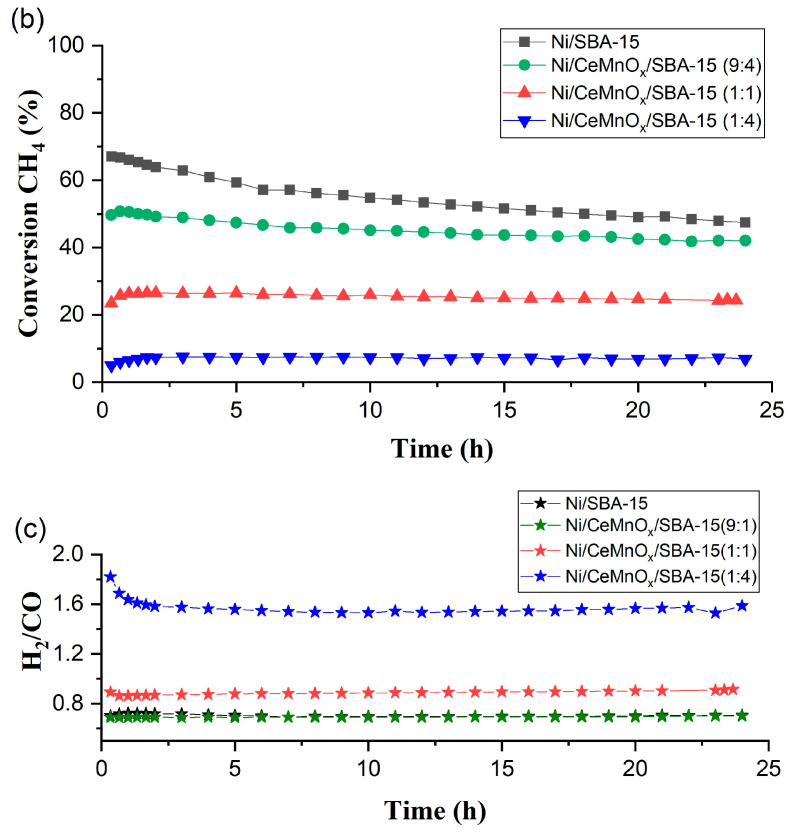
Long-run DRM tests at 650 °C during 24 h: CO_2_ conversions (**a**), CH_4_ conversions (**b**), H_2_/CO ratio (**c**).

Catalyst stability tests in DRM were carried out for 24 h (Figure 8), and for Ni/CeMnO_x_/SBA-15 (9:1) a test during 80 h was also performed (Figure S5). The Ni/SBA-15 was the most active catalyst at 650 °C, and the initial conversion of CH_4_ and CO_2_ was 68 and 80%, respectively, which is 34% and 25% higher than in the case of gradient temperature test (Figure 8). However, high conversions of the reagents over Ni/SBA-15 are accompanied by the accumulation of significant amount of carbonaceous deposit on the surface of the unmodified sample (42.2% within 24 h, Table 4). For the modified (9:1 and 1:1) Ni catalysts almost complete suppression of the process of carbon deposition on the surface is observed while maintaining high values of the “useful” conversion of reagents (9:1) without a significant decrease in activity during the 24 h long test. A long run test up to 80 h for the most promising sample, Ni/CeMnO_x_/SBA-15 (9:1) showed a slight decrease in the conversion of reagents while maintaining the H_2_/CO ratio remains constant to 0.7, confirming the value registered after 24 h of reaction.

Among the catalysts based on modified supports, the highest conversion is observed for Ni/CeMnO_x_/SBA-15 (9:1) and amount to 51 and 69% for CH_4_ and CO_2_, respectively. In addition, this catalyst exhibits greater stability compared with the unmodified Ni/SBA-15. Thus, over 24 h, the conversion of CH_4_ on the Ni/SBA-15 catalyst decreased by 30% compared to the initial value, and on the Ni-Ni/CeMnO_x_/SBA-15 (9:1) catalyst it dropped by 18%.

For the Ni/CeMnO_x_/SBA-15 (1:4) and Ni/CeMnO_x_/SBA-15 (1:1) catalysts, the reagent conversions remain constant for 24 h (Figure 8, Table 4). The stability of the modified catalysts is owing to the enhancement of the metal–support interactions, as shown by the results of Raman spectroscopy and TPR-H_2_. The oxygen vacancies in the presence of Ce and Mn oxides should also affect the oxidation of the resulting carbon and prevent carbon deposition. Optimization of the ratio between the amount of CeMnO_x_ modifier and active component in the SBA-15 structure is a promising direction for creating highly efficient and stable DRM catalysts.

Simultaneously with a decrease in the reagent conversions with a rise in the Mn content in the modified support, an increase in the H_2_/CO ratio is also observed, reaching values of 1.64–1.58 for the Ni/CeMnO_x_/SBA-15 (1:4) sample (Figure 8, Table 4). We have previously found a similar decrease in activity with high H_2_/CO > 1 values for supported catalysts in the presence of a significant amount of manganese on lanthanum manganites [46] and Ni/LaMnO_x_/SBA-15 (1:1) (Figure S6). An increase in this ratio indicates a decrease in the contribution of the reverse WGS (CO_2_ + H_2_O ↔ CO + H_2_O) as well as the thermodynamically possible hydrogenation of carbon dioxide (CO + H_2_ ↔ C + H_2_O) [47]. In addition, the decrease in CO content in the products may be due to its oxidation by oxide forms of manganese [48].

Manganese additives were previously considered as modifiers for DRM catalysts to prevent coking [23,49,50]. However, the data on the activity of such catalysts are rather contradictory. Thus, in work [38], an improvement in the characteristics of Ni-Mn/SiO_2_ was shown to be associated with better dispersion of the active component due to the formation of NiMn_2_O_4_. This was believed to enhance the interaction of the support with Ni and Mn_2_SiO_4_, which in turn limited the migration and aggregation of metallic Ni particles. At the same time, Li et al. [43] showed a positive effect of Mn-related phases on the catalytic characteristics of Ni/MnO-Al_2_O_3_ in DRM processes. Thus, the introduction of 15 wt.% MnO on Al_2_O_3_ led to an increase in the dispersion of Ni particles and a decrease in the activity of the catalyst due to the decoration of MnO_x_ active sites. The basicity of MnO promoted CO_2_ adsorption, which can reduce carbon formation by CO disproportionation owing to the shifting of the equilibrium. In addition, the formation of manganese carbonate at the manganese oxide–nickel interface facilitated the removal of active CH_x_ adsorbed intermediates formed on the surface of catalyst prior to their conversion into non-reactive carbon. However, significant decoration can lead to a decrease in the methane conversion.

In the case of Ni/CeMnO_x_/SBA-15 (1:4), we can also assume a similar decoration mechanism based on the TPR-H_2_, SAXS and STEM data. Probably, manganese modifier particles on the surface of the SBA-15 hydrophobic support are more mobile and reactive compared to MnO particles in the Ni/CeMnO_x_ (1:4) double oxide structure [23], which leads to a more significant blocking of the active sites and an activity drop (Figure 8, Table 4).

### 3.4. Characterization of Spent Catalyst

#### 3.4.1. TGA Study after Catalytic Tests

To assess the accumulation of C-containing deposits on the catalyst surfaces after stability tests, studies were carried out by the TGA method under air flow (Figure S7). The TG curves for all samples contain a slight weight gain in the range of 250–500 °C, from 1.0 wt.% for Ni/SBA-15 to up to 2.5 wt.% for Ni/CeMnO_x_/SBA-15 (9:1). The increase in the mass of the spent samples can be explained by the oxidation of Ni^0^ NPs for the Ni/SBA-15 catalyst and the concomitant oxidation of Ni^0^ along with some reduced CeMnO_x_ species in the case of catalysts on the modified support. For the Ni/SBA-15 catalyst, the oxidation of the carbon coke accumulated during the long run test begins at temperatures above 480 °C, and the weight loss corresponds to 42.2 wt.%. The introduction of modifiers on the support structure essentially reduces the carbonization of the catalysts within 24 h. For the Ni/CeMnO_x_/SBA-15 (9:1) catalyst, which exhibits the highest activity among the modified samples, the weight loss for carbon removal is 2.7 wt.% (Figure S7, green line). The same catalyst, after 80 h of reaction, exhibited a weight loss of 20.6 wt.%. No evident mass change occurs for Ni/CeMnO_x_/SBA-15 (1:4) and for Ni/CeMnO_x_/SBA-15 (1:1), so for such catalysts the absence of carbon deposit is associated with both lower methane conversion and the presence of oxygen vacancies.

#### 3.4.2. XRD Study after Catalytic Tests

**Figure 9:**
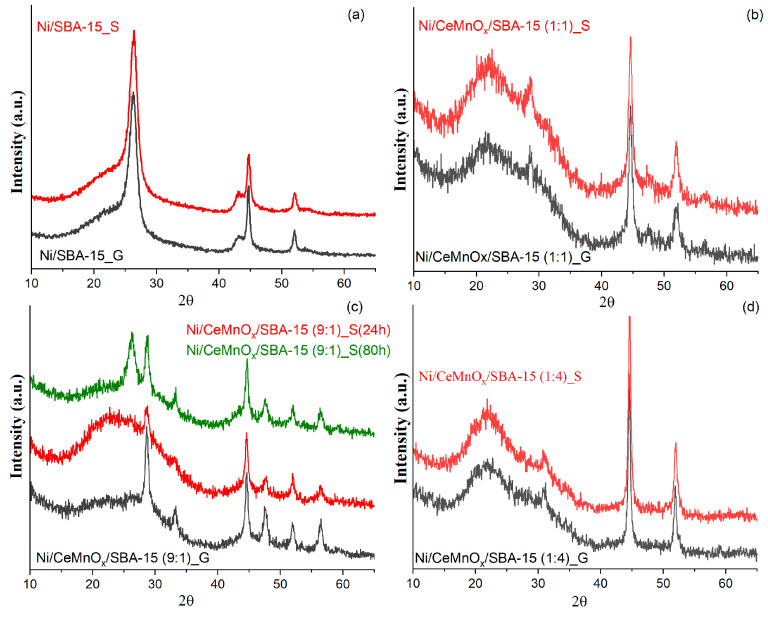
XRD patterns for spent catalysts after gradient temperature tests (black) and long-run tests during 24 h (red) or 80 h (green) for Ni/SBA-15 (**a**), Ni/CeMnO_x_/SBA-15 (1:1) (**b**), Ni/CeMnO_x_/SBA-15 (9:1) (**c**), Ni/CeMnO_x_/SBA-15 (1:4) (**d**).

Figure 9 exhibits the XRD results for the spent catalysts after gradient and long-run tests. All spent catalysts exhibit diffraction reflections at 2θ = 23° and 2ϴ = 44.5° and 52° corresponding to the SiO_2_ and Ni NP phase, respectively. The reflexes for NiO NPs were observed for spent Ni/SBA-15 (a shoulder at 43°) only, thus indicating that the active Ni NPs were oxidized to inactive NiO under the action of CO_2_ and H_2_O as oxidizing agents [51]. The diffraction reflections at 26.3°, which are observed only for Ni/SBA-15 (Figure 9a) and Ni/CeMnO_x_/SBA-15 (9:1) after 80 h test (Figure 9c green line), are attributed to graphite carbon deposition. The sizes of Ni crystallites were calculated using the Scherrer equation (Table 4). As can be seen, only for Ni/CeMnO_x_/SBA-15 (1:4), an increase in Ni crystallite sizes due to sintering (from 13 to 18 nm, and to 21 nm for gradient and long-run tests, respectively) is observed. As for the other catalysts, the sizes of Ni crystallites after the temperature gradient test remain practically unchanged, and after a long run, the crystallites decrease due to redispersion.

#### 3.4.3. Raman Spectroscopy

**Figure 10:**
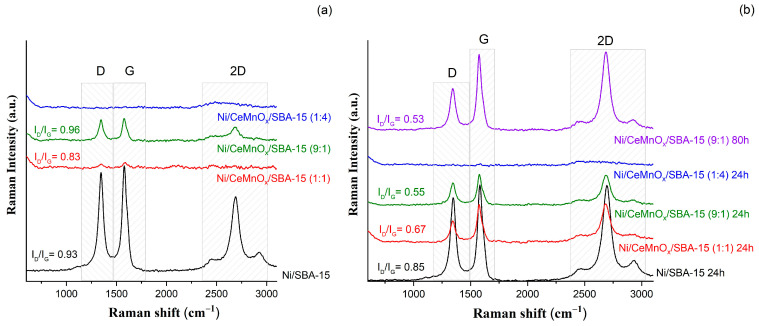
Raman spectra for the spent catalysts after the gradient (**a**) and long-run (**b**) tests.

Figure 10 exhibits the Raman spectra for the spent catalysts after the gradient (panel (a)) and long-run (panel (b)) tests. Raman spectra refer to spectra of multiwalled carbon nanotubes (MWCNT) deposited on quartz support. The spectra are seen to contain the G band at 1590 cm^−1^, which is attributed to almost all carbon nanoforms. The D band observed at 1320 cm^−1^ pointed at some disorder in the graphene structure, while the 2D and (D + G) 2G peaks seen at 2700 cm^−1^ and 2900 cm^−1^ are known to be overtones of the G band [52]. For Ni/CeMnOx/SBA-15 (1:4) the carbon was not observed after gradient test as well as for long-run test. For other spent catalysts the G band is more intensive than D band, that means a higher contribution of the graphitized carbon formed. Combination of the G band intensity with the ratio I_D_/I_G_ is known to give information on the defect density. Typically, lower I_D_/I_G_ values indicate higher crystallinity resulted from the formation of graphitized carbon. The estimated I_D_/I_G_ ratio for catalysts after gradient temperature test follows the order 0.96 > 0.93 > 0.83 for the Ni/CeMnOx/SBA-15 (9:1), Ni/SBA-15 and Ni/CeMnO_x_/SBA-15 (1:1), respectively. The ID/IG ratio for samples after stability test the following order 0.85 > 0.67 > 0.55 > 0.53 for Ni/SBA-15, Ni/CeMnO_x_/SBA-15 (1:1), Ni/CeMnO_x_/SBA-15 (9:1) and Ni/CeMnO_x_/SBA-15 (9:1) after 80 h, respectively.

Increase in reaction time was observed to lead to a higher degree of crystallinity (after stability test during 24 h and, in the case of sample Ni/CeMnO_x_ /SBA-15 (9:1), as well as during 80 h) in comparison with the sample subjected to gradient test. Such an increase in crystallinity is much more pronounced for samples Ni/CeMnO_x_/SBA-15 (9:1) and Ni/CeMnO_x_ /SBA-15 (1:1).

Thus, Raman studies show that the type and fractions of different carbon deposits on the surface of spent Ni catalysts are dependent on the nature of support. On the surface of unmodified Ni/SBA-15 catalyst, carbon deposits coated the surface of Ni NPs, which led to the growth of graphite-like carbon. In the case of catalyst Ni/CeMnO_x_/SBA-15 (9:1), coke layers do not cover active Ni species after testing the sample for both 24 h or 80 h.

## 4. Conclusions

Ni catalysts for dry reforming of methane have been prepared over CeMnO_x_-modified SBA-15 and characterized using several physical-chemical methods. The main effect of modifying SBA-15 with cerium and manganese oxides is the decreased size of NiO particles and the enhanced interaction between NiO and the support, as confirmed by the higher reduction temperature of Ni^2+^ species with respect to the unpromoted catalyst. Such effects influence the catalytic conversion of CH_4_ and CO_2_ that, at 650 °C, during long-run tests, varied as follows: Ni/SBA-15 > Ni/CeMnO_x_/SBA-15 (9:1) > Ni/CeMnO_x_/SBA-15 (1:1) > Ni/CeMnO_x_/SBA-15 (1:4). However, although Ni/SBA-15 exhibits the best conversion (up to 650 °C in the gradient tests and during the first 12 h of reaction at 650 °C), in the case of Ni/ CeMnO_x_/SBA-15 modified catalysts an increase in the Ni dispersion, thanks to the strong interaction with modifiers, has a positive impact on the catalyst stability. The best catalytic performance is achieved at the Ce/Mn ratio of 9:1, i.e., the CH_4_ and CO_2_ conversions at 650 °C are 51 and 69%, respectively. Moreover, in 24 h stability test, the CH_4_ conversion over Ni/CeMnO_x_/SBA-15 (9:1) decreases by 18 rel.% as compared to a 30 rel.% decrease for the unmodified Ni/SBA-15 catalyst.

A long-run test at 650 °C up to 80 h was carried out for the most promising sample Ni/CeMnO_x_/SBA-15 (9:1) showed a slight decrease in the conversion of reagents, while maintaining the H_2_/CO ratio constant to 0.7, confirming the value registered after 24 h of reaction. The amount of carbon accumulated on such a promising catalyst corresponded to a weight loss of 20.6 wt.% after 80 h in comparison with 42.2 wt.% for the undoped Ni/SBA-15 after only 24 h of reaction at the same temperature.

In all the Ni/CeMnO_x_/SBA-15 samples oxygen vacancies likely due to the replacement of Ce^4+^ by Mn^n+^ were detected along with the formation of Ce^3+^. Ni/CeMnO_x_/SBA-15 (9:1) and Ni/CeMnO_x_/SBA-15 (1:1) exhibited the highest concentration of surface oxygen vacancies that reduce the formation of carbon phase during the reaction, thereby improving their stability.

The results here reported can be used to prepare the next generations of effective DRM catalysts. Optimization of the ratio between the amount of CeMnO_x_ modifier and active component in the SBA-15 structure is a promising direction for creating highly efficient and stable DRM catalysts.

## Figures and Tables

**Table 1:** Element content by XRF data.

Samples	Content, wt.%			Ce/Mn, mol.%
	MnO_2_	CeO_2_	NiO	
Ni/SBA-15	-	-	10.3	
Ni/CeMnO_x_/SBA-15 (1:1)	4.7	8.6	10.6	0.93
Ni/CeMnO_x_/SBA-15 (9:1)	1.1	16.8	11.6	9.3
Ni/CeMnO_x_/SBA-15 (1:4)	7.7	3.6	12.5	0.24

**Table 2:** Textural properties and structural parameters of calcined SBA-based supports and Ni catalysts.

Sample	S_BET_, m^2^/g	V_pore_, cm^3^/g	Pore Size, nm	d(100) ^1^, nm	*a* ^2^, nm	D(NiO) ^3^, nm	D(Ni) ^3^, nm
SBA-15	763	0.98	6.1	9.28	10.72	-	-
CeMnOx/SBA-15 (1:1)	547	0.75	6.2	9.07	10.47	-	-
CeMnOx/SBA-15 (9:1)	525	0.66	6.1	9.11	10.52	-	-
CeMnOx/SBA-15 (1:4)	565	0.80	6.1	9.13	10.54	-	
Ni/SBA-15	494	0.70	5.8	8.85	10.22	18	19
Ni/CeMnOx/SBA-15 (1:1)	318	0.47	5.7/5.0	8.87	10.24	10	17
Ni/CeMnOx/SBA-15 (9:1)	294	0.41	5.7/4.8	8.76	10.12	8	16
Ni/CeMnOx/SBA-15 (1:4)	363	0.57	5.6	8.86	10.23	9	13

**^1^** Plane distance d(100) was computed according to Bragg’s law (λ = 2d sinθ). **^2^** Lattice parameter (*a*) for pores with a hexagonal structure from SAXS data calculated as *a* = 2d(100)√3. **^3^** Particle size (D) from XRD data.

**Table 3:** Reduction temperatures and hydrogen consumption by supports and catalysts.

Sample	T_max_ (°C)	Reactions	Experimental H_2_ Consumptions (mmol/g) *	Theoretical H_2_ Consumptions (mmol/g) **
CeMnO_x_/SBA-15 (9:1)	240–750	Ce^4+^ → Ce_3+_	0.47	―
CeMnO_x_/SBA-15 (1:1)	290–525	Mn^4+^/Mn^3+^ → Mn^2+^	0.38	―
	240–750	Ce^4+^ → Ce^3+^		
CeMnO_x_/SBA-15 (1:4)	280–570	Ce^4+^ → Ce_3+_	0.46	―
Ni/SBA-15	390–730	Ni^2+^ → Ni^0^	1.41	1.77
Ni/CeMnO_x_/SBA-15 (9:1)	230–380	Ni^2+^ → Ni^0^	1.54	1.55
Ni/CeMnO_x_/SBA-15 (1:1)	290–410	Ni^2+^ → Ni^0^	1.79	1.51
		Mn^4+^/Mn^3+^ → Mn^2+^		
	410–480	Ni^2+^ → Ni^0^		
		Mn^3+^ → Mn^2+^		
	480–817	Ni^2+^ → Ni^0^		
Ni/CeMnO_x_/SBA-15 (1:4)	300–380	Ni^2+^ → Ni^0^	1.61	1.67
		Mn^4+^/Mn^3+^ → Mn^2+^		
	380–500	Ni^2+^ → Ni^0^		
		Mn^3+^ → Mn^2+^		
	500–800	Ni^2+^ → Ni^0^		

* Total H_2_ consumption of both modifier oxides and NiO. ** Calculation for real NiO content by XRF data.

**Table 4:** Performance on catalysts in temperature and stability tests.

Catalytic Performance			Catalysts		
		Ni/SBA-15	Ni/CeMnO_x_/SBA-15 (9:1)	Ni/CeMnO_x_/SBA-15 (1:1)	Ni/CeMnO_x_/SBA-15 (1:4)
Conversion (X, %) and H_2_/CO at 650 °C in gradient temperature test	X(CH_4_)	32	40	29	8
	X(CO_2_)	55	55	52	17
	H_2_/CO	0.70	0.72	0.73	1.2
Conversion (X, %) and H_2_/CO at 650 °C in stability test	X(CH_4_) ^1 h^	66	51	26	7
	X(CO_2_) ^1 h^	80	69	50	27
	H_2_/CO ^1 h^	0.72	0.69	0.86	1.64
	X(CH_4_) ^24 h^	47	42	24	7
	X(CO_2_) ^24 h^	68	60	50	21
	H_2_/CO ^24 h^	0.71	0.70	0.91	1.59
C weight loss, %		42.2	2.7	-	-
Ni^0^ particle size (nm) (after g/s tests)		19/17	15/12	16/10	18/21

g: gradient temperature test; s: stability test; 1 h: performance after 1 h of catalyst operation; 24 h: performance after 24 h of catalyst operation; C weight loss, %, evaluated by TGA (Figure S7), due to the oxidation of carbon accumulated at 650 °C during the stability test; Ni particle size (nm) after gradient (g) and stability (s) test was calculated by XRD.

## Data Availability

Data presented in this work are available on request from the corresponding authors.

## Supplementary Materials

The following supporting information can be downloaded at: https://www.mdpi.com/article/10.3390/nano13192641/s1, Figure S1: SAXS patterns for SBA-15, calcined at 500 and 800 °C; Figure S2: UV–vis diffuse reflectance spectra for CeMnO_x_/SBA-15 with different Ce/Mn molar ratios (**a**) and the corresponding Ni/CeMnO_x_/SBA-15 catalysts on the basis thereof (**b**); Figure S3: HR TEM of Ni/CeMnOx/SBA-15 (1:4) catalyst reduced at 750 °C; Figure S4: Particle size distribution of calcined and reduced Ni catalysts (**a**) Ni/SBA-15, (**b**) Ni/CeMnOx/SBA-15 (1:1) based on SAXS data; Figure S5: Long-run DRM tests on Ni/CeMnO_x_/SBA-15 (9:1) at 650 °C during 80 h; Figure S6: Temperature dependences of CH_4_ and CO_2_ conversions and H_2_/CO ratio for Ni/LaMnO_x_/SBA-15 (1:1) catalyst. Filled symbols—heating, empty symbols—cooling; Figure S7: TGA for samples after 24 h of time on stream. Black, blue, red and green colors correspond to Ni/SBA-15, Ni/CeMnO_x_/SBA-15 (1:4), Ni/CeMnO_x_/SBA-15 (1:1), Ni/CeMnO_x_/SBA-15 (9:1). The green dashed line refers to the long run after 80 h.
