# Supplementary material for: Ni-Based SBA-15 Catalysts Modified with CeMnOx for CO2 Valorization via Dry Reforming of Methane: Effect of Composition on Modulating Activity and H2/CO Ratio
Source: Nanomaterials (Basel). 2023 Sep 26;13(19):2641. doi: 10.3390/nano13192641 (PMC10574277; doi:10.3390/nano13192641)
Supplement: Supplementary file 1 [file nanomaterials-13-02641-s001.zip › nanomaterials-2579109-supplementary.pdf]

nanomaterials

MDPI

Supporting information

Ni-Based SBA-15 Catalysts Modified with $CeMnO_x$ for CO_2 Valorization via Dry Reforming of Methane: Effect of Composition on Modulating Activity and H_2/CO Ratio

Maria V. Grabchenko [1], Natalia V. Dorofeeva [1], Valery A. Svetlichnyi [2], Yurii V. Larichev [3], Valeria La Parola [4], Leonarda Francesca Liotta [4,*], Sergei A. Kulinich [5,*] and Olga V. Vodyankina [1]

1. Laboratory of Catalytic Research, Tomsk State University, 634050 Tomsk, Russia; marygra@mail.ru (M.V.G.); natalia10@mail.ru (N.V.D.); vodyankina_o@mail.ru (O.V.V.)
2. Laboratory of Advanced Materials and Technology, Siberian Physical Technical Institute, Tomsk State University, 634050 Tomsk, Russia; v_svetlichnyi@bk.ru (V.A.S.)
3. Boreskov Institute of Catalysis SB RAS (BIC SB RAS), 630090 Novosibirsk, Russia; ylarichev@gmail.com
4. Institute for the Study of Nanostructured Materials (ISMN), National Research Council (CNR), 90146 Palermo, Italy; valeria.laparola@cnr.it
5. Research Institute of Science & Technology, Tokai University, Hiratsuka, Kanagawa 259-1292, Japan

* Correspondence: leonardafrancesca.liotta@cnr.it (L.F.L.); skulinich@tokai-u.jp (S.A.K.)

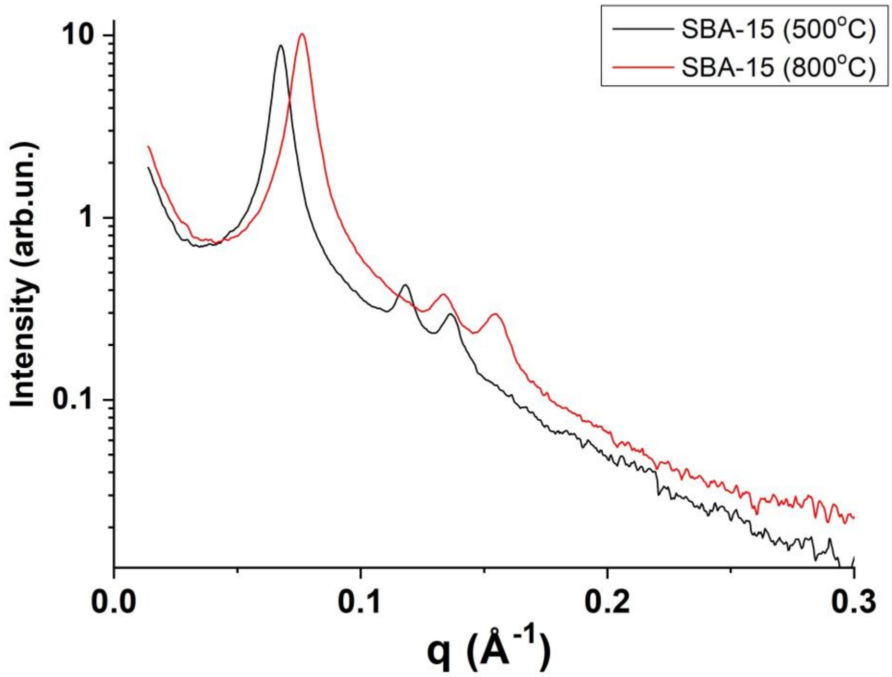

Figure S1. SAXS patterns for SBA-15, calcined at 500 and 800 °C.

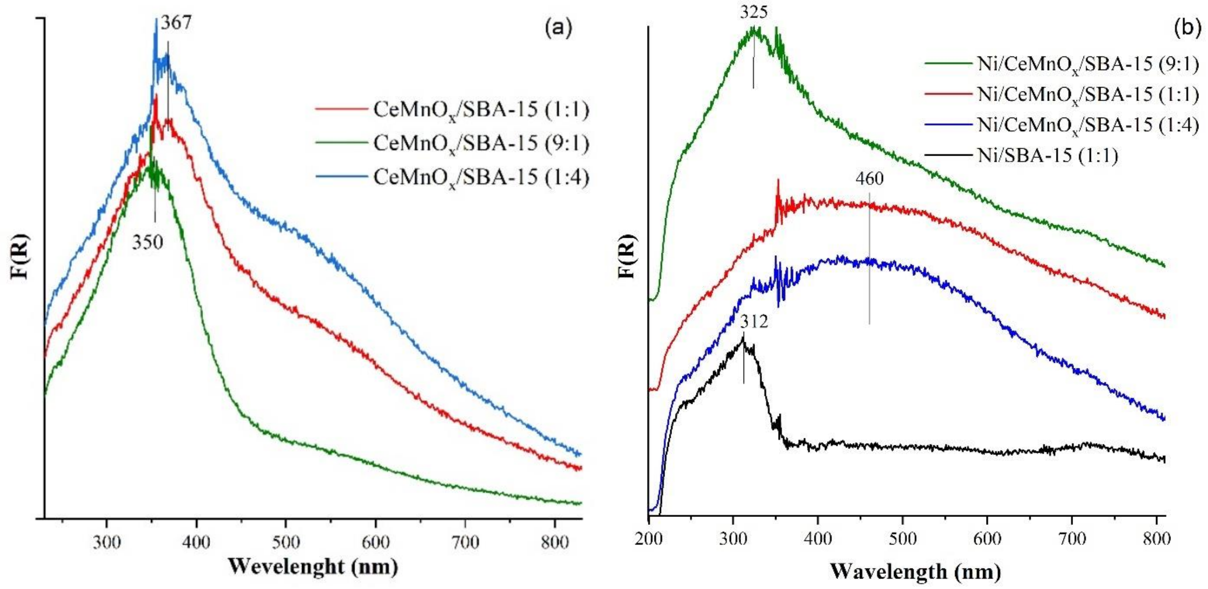

Figure S2. UV–vis diffuse reflectance spectra for $CeMnO_x$/SBA-15 with different Ce/Mn molar ratios (a) and the corresponding Ni/$CeMnO_x$/SBA-15 catalysts (b).

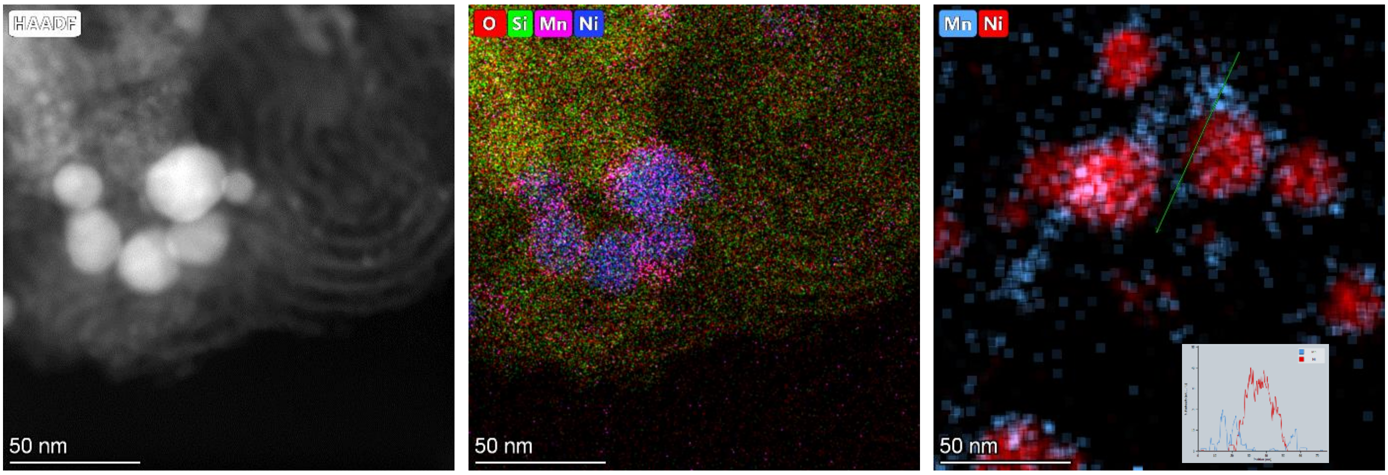

Figure S3. HR TEM of Ni/$CeMnO_x$/SBA-15 (1:4) catalyst reduced at 700 ºC

(a)

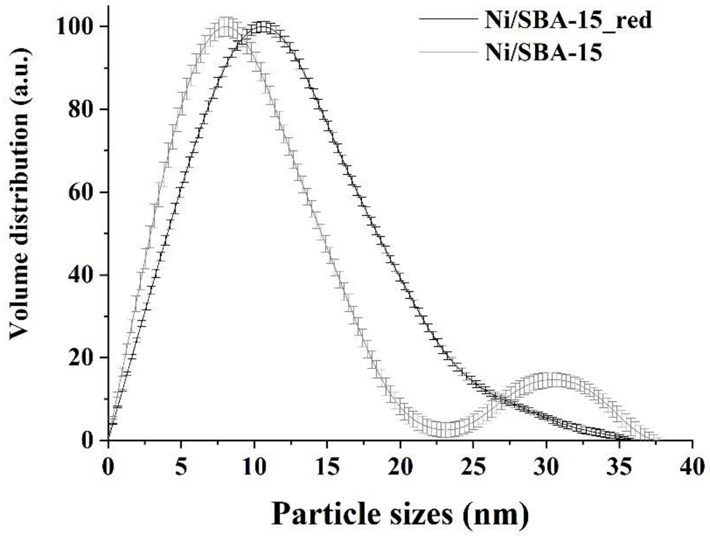

(b)

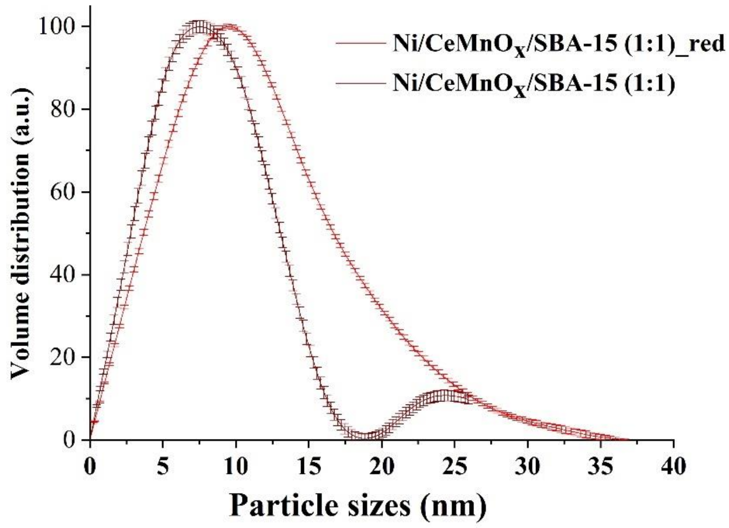

Figure S4. Particle size distribution of calcined and reduced Ni catalysts (a) Ni/SBA-15, (b) Ni/CeMnO$_x$/SBA-15 (1:1) based on SAXS data.

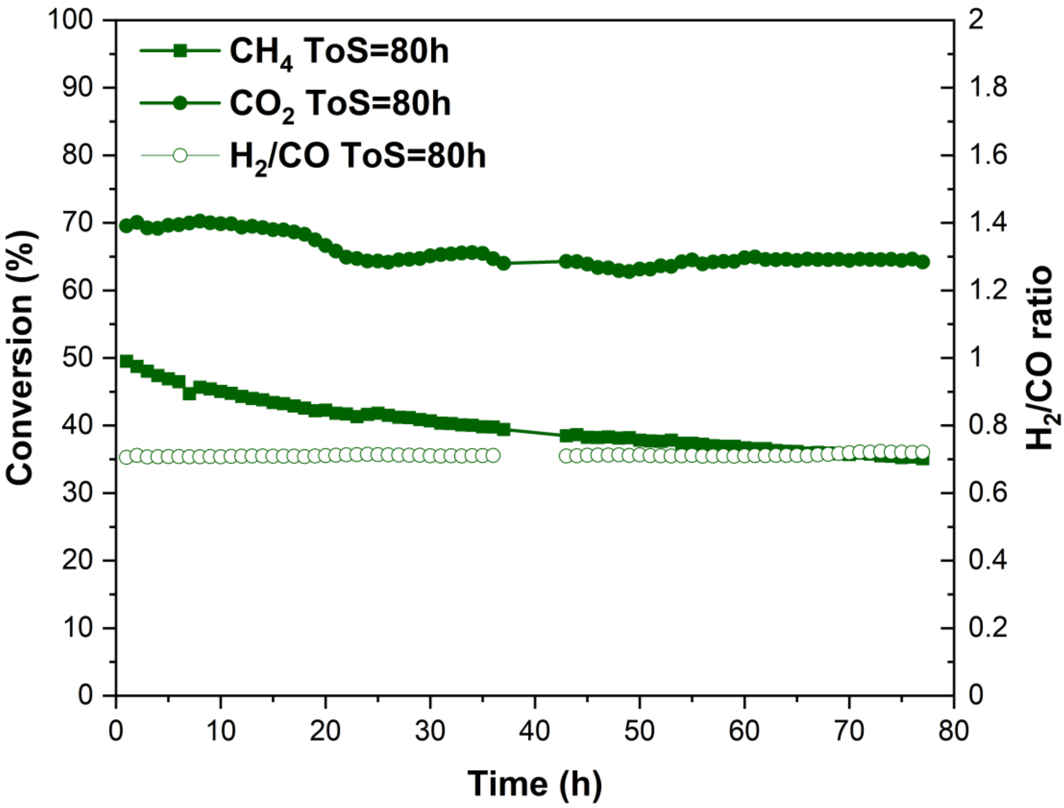

Figure S5. Long-run DRM tests on Ni/CeMnO$_x$/SBA-15 (9:1) at 650 °C during 80 h time on stream (ToS).

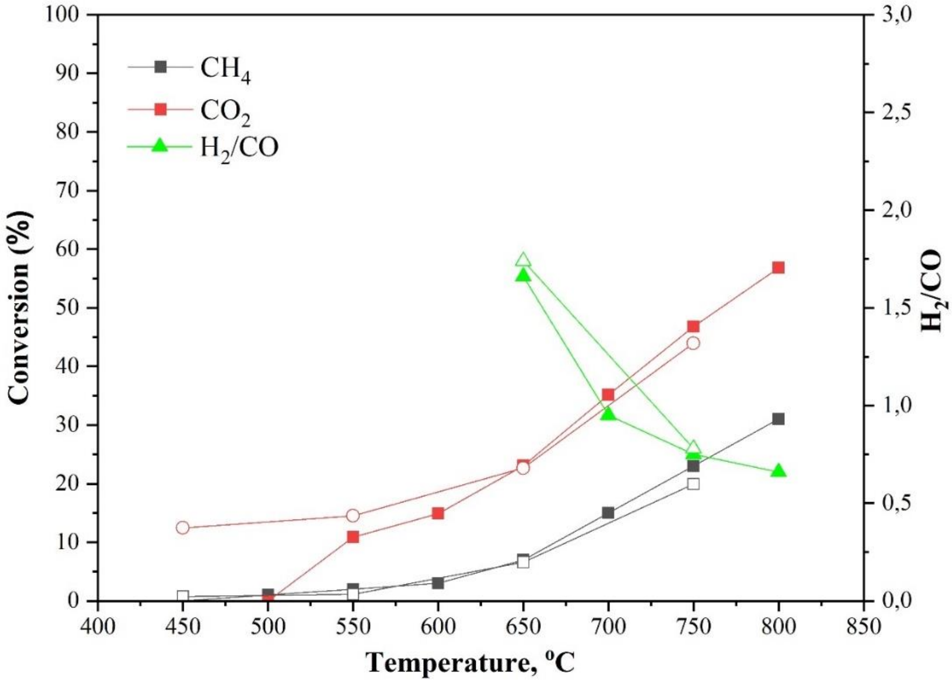

Figure S6. Temperature dependences of CH_4 and CO_2 conversions and H_2/CO ratio for Ni/LaMnO$_x$/SBA-15 (1:1) catalyst. Filled symbols – heating, empty symbols – cooling.

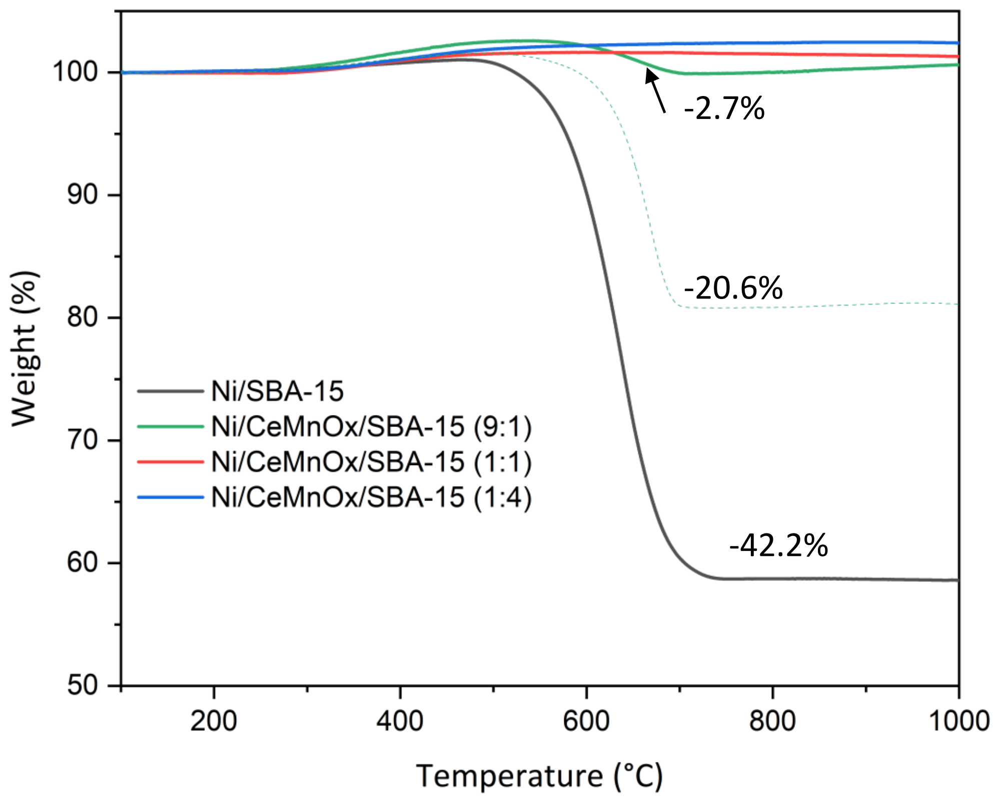

Figure S7. TGA for samples after 24 h of time-on-stream. Black, blue, red, and green colors correspond to Ni/SBA-15, Ni/CeMnO$_x$/SBA-15 (1:4), Ni/CeMnO$_x$/SBA-15(1:1), Ni/CeMnO$_x$/SBA-15 (9:1). The green dashed line refers to the long-run on Ni/CeMnO$_x$/SBA-15 (9:1) at 650 ^{o}C during 80 h.
